# Supplementary material for: Quantized classical response from spectral winding topology
Source: Nat Commun. 2021 Sep 6;12:5294. doi: 10.1038/s41467-021-25626-z (PMC8421445; doi:10.1038/s41467-021-25626-z)
Supplement: Supplementary file 1 — Supplementary Information [file 41467_2021_25626_MOESM1_ESM.pdf]

Supplementary Information - Quantized Classical Response From Spectral Winding Topology

Linhu Li,[1,*] Sen Mu,[2] Ching Hua Lee,[2,†] and Jiangbin Gong[2,‡]

[1]Guangdong Provincial Key Laboratory of Quantum Metrology and Sensing & School of Physics and Astronomy, Sun Yat-Sen University (Zhuhai Campus), Zhuhai 519082, China

[2]Department of Physics, National University of Singapore, Singapore 117551, Republic of Singapore

(Dated: August 20, 2021)

SUPPLEMENTARY NOTE 1: CLASSICAL VS. QUANTUM RESPONSE

In this Supplementary Note 1, we briefly describe the concept of quantized linear response in a quantum setting, as a comparison with the classifical quantized response we define. Consider the quantum setting described by a Hamiltonian $\hat{H}(t) = \hat{H}_0 + \epsilon(t)\hat{H}'$, where $\hat{H}_0$ is the equilibrium Hamiltonian, $\hat{H}'$ the perturbed operator and $\epsilon(t)$ a time-dependent factor that controls the perturbation strength. The linear response of a chosen operator $\hat{\phi}$ (which has zero expectation when $\epsilon(t) = 0$) is its expectation $\hat{\phi}(t)$ after $\epsilon(t)$ is switched on. In frequency space, we write

$$\langle\hat{\phi}\rangle(\omega) = D(\omega)\epsilon(\omega), \tag{1}$$

where

$$\begin{aligned} D(\omega) &= -i\int_0^\infty \langle[\hat{\phi}(t), \hat{H}'(0)]\rangle\, dt \\ &= -iZ^{-1}\int_0^\infty \mathrm{Tr}\left(e^{-H_0/(k_BT)}[\hat{\phi}(t), \hat{H}'(0)]\right) dt \\ &= \frac{1}{Z}\sum_{nm}\frac{\langle n|\hat{\phi}|m\rangle\langle m|\hat{H}'|n\rangle(e^{-E_n/(k_BT)} - e^{-E_m/(k_BT)})}{\omega + i\delta - (E_m - E_n)} \end{aligned} \tag{2}$$

where $Z = \mathrm{Tr}\left(e^{-\hat{H}_0/(k_BT)}\right)$, k_B is the Boltzmann constant and T is the temperature, and the trace is taken over all eigenstates $|n\rangle$ satisfying $\hat{H}_0|n\rangle = E_n|n\rangle$. Very importantly, it has been assumed that the system is in a ground state where the eigenstates $|n\rangle$ are occupied according to the Boltzmann probability distribution $\propto e^{E_n/(k_BT)}$. By expressing the trace as a momentum-space integral which shall correspond to a topological invariant, a topologically quantized response coefficient can be obtained, for instance, with the DC limit of $D(\omega)/i\omega$ in the case of Hall response.

By contrast, Supplementary Eq. 2 (the Kubo formula) is inapplicable in classical settings, where the systems do not settle into a ground state. As discussed in the main text, an external coherent drive $\boldsymbol{\epsilon}(t) = \boldsymbol{\epsilon}(\omega)\exp(-i\omega t)$ (in the frequency domain) and its resultant classical response field $\boldsymbol{\phi}(t) = \boldsymbol{\phi}(\omega)\exp(-i\omega t)$ can be related by the Green's function matrix G,

$$\boldsymbol{\phi}(\omega) = G(\omega,\gamma)\boldsymbol{\epsilon}(\omega),\ G(\omega,\gamma) = \frac{1}{\omega + i\gamma - H}, \tag{3}$$

analogous to Supplementary Eq. 2 which is exclusively for quantum settings.

SUPPLEMENTARY NOTE 2: CASES WITH COUPLINGS ACROSS DIFFERENT RANGES

In the main text, we have already witnessed an intriguing example where both nearest-neighboring and next-nearest-neighbor couplings present in the system. In that case, with respect to different reference energy points, the spectral winding can have different nonzero values and this is also manifested as different quantized responses. Benchmarking this with our decoupled subchain picture, this indicates that there is competition in different regimes, and the underlying topological robustness associated with either single-chain physics or two coupled subchain physics still yields, remarkably, quantized responses. The next question is then, if we add more and more complexity to a non-Hermitian lattice system with many coexisting hopping length scales, can we still observe quantization and

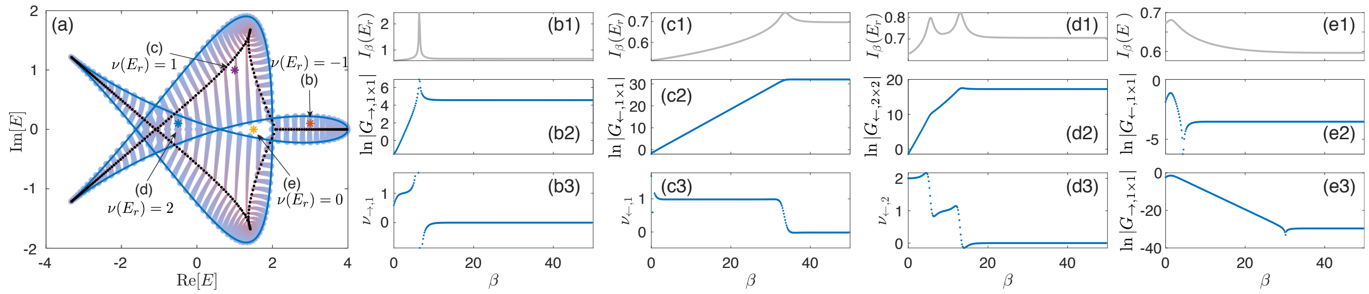

FIG. S1. (a) PBC (blue loop) and OBC (black dots) spectra of a system with many coexisting hopping length scales, and the PBC-OBC spectral evolution between them (blue-purple curves), tuned by the parameter β. Four reference energy values E_r are chosen to inspect the possible quantization of the gradient of the logarithm of directional amplification with respect to β, each with a different winding number $\nu(E_r)$, as indicated by the four colored marks. (b)-(e) show the summed reverse energy spacing $I_\beta(E_r)=\sum_n |1/(E_n(\beta)-E_r)|$, logarithm of the amplification ratio for the block of G associated with the winding number $\nu(E_r)$, as well as $\nu_{\leftarrow,m}$ and $\nu_{\rightarrow,m}$, as functions of β. In (e) we have $\nu(E_r)=0$, hence we only illustrate amplification ratios $G_{\leftarrow,1\times1}$ and $G_{\rightarrow,1\times1}$, which are both less than unity, indicating no amplification for a signal moving either toward the left or the right. Parameters are $t_2=2$, $t_{-2}=1$, $t_3=0$, $t_{-3}=1$, and $t_{\pm m}=0$ for all other values of m, with $N=300$ lattice sites.

hence a clear correspondence between spectral winding and the signal amplification. Our answer is yes based on more computational tests.

Consider then a lattice model with nonzero terms $\{t_{-r},t_{-r+1},...,t_{l-1},t_l\}$, and none of them is dominating over the rest. We can still view this system as one comprised of m subchains, with $m\leqslant \mathrm{Max}[r,l]$. We can investigate if the same response functions $\nu_{\leftarrow,m}$ or $\nu_{\rightarrow,m}$ can reflect the spectral winding behaviors. To proceed specifically, consider the following Hamiltonian as an example,

$$H=\sum_{x=1}^{N}\sum_{j=-r}^{l} t_j \hat{c}_j^\dagger \hat{c}_{x+j}, \tag{4}$$

with $r=l=3$, and the boundary coupling tuned via $t_j\to e^{-\beta}t_j$ when it connects sites at different ends of this 1D chain. The rather complicated spectral winding behavior is shown in Fig. S1(a), indicating winding numbers -1, 1, 2, and 0. As shown in Fig. S1(b)-(d), the obtained $\nu_{\leftarrow,m}$ or $\nu_{\rightarrow,m}$ for $m=1$ or $m=2$ still shows relatively clear plateaus for cases with nonzero $\nu(E_r)$, with the transitions of these plateaus in excellent agreement with the critical β values for which the spectral winding numbers make jumps. In panel (c3), the quantization in $\nu_{\leftarrow,1}$ is clearly seen. A careful reader might notice that in panel (b3) and (d3), the plateaus of the obtained response function $\nu_{\rightarrow,1}$ are so not clearly quantized. To double check if this is merely a finite-size effect, we have increased the size of the model system and then much better quantized plateaus are indeed observed, as presented in Fig. S2. As to the case of $\nu(E_r)=0$ labeled by the yellow star in Fig. S1(a), no amplification is obtained for a signal moving toward either the left- or the right-hand side, as indicated by the always-negative $\ln|G_{\leftarrow,1\times1}|$ and $\ln|G_{\rightarrow,1\times1}|$ in Fig. S1(e).

SUPPLEMENTARY NOTE 3: QUANTIZED RESPONSE AGAINST DISORDER

As the spectral winding topology of the non-Hermitian Hamiltonian does not depend on the system's symmetries, we expect the quantized plateaus to be robust against disorder without any symmetry restriction. In Fig. S3 we illustrate the effect of certain disorder for the model of Supplementary Eq. 4 with $t_1=1$, $t_{-1}=0.5$, $t_2=2$, and $t_m=0$ for every other m, i.e. the example model of Figs. 1 and 2 in the main text. Adding to this model, we consider some extra disorder terms given by

$$H_{\text{disorder}}=\sum_{x=1}^{N}\sum_{j=-2}^{2} W_j(x)\hat{c}_x^\dagger \hat{c}_{x+j}, \tag{5}$$

with $W_j(x)$ a random value between W and $-W$. It is seen that a nonzero W may change the sharpness of the jump of $\nu_{\rightarrow,2}$, as the spectral evolution may not pass a chosen reference energy E_r exactly in a finite-size numeric; and

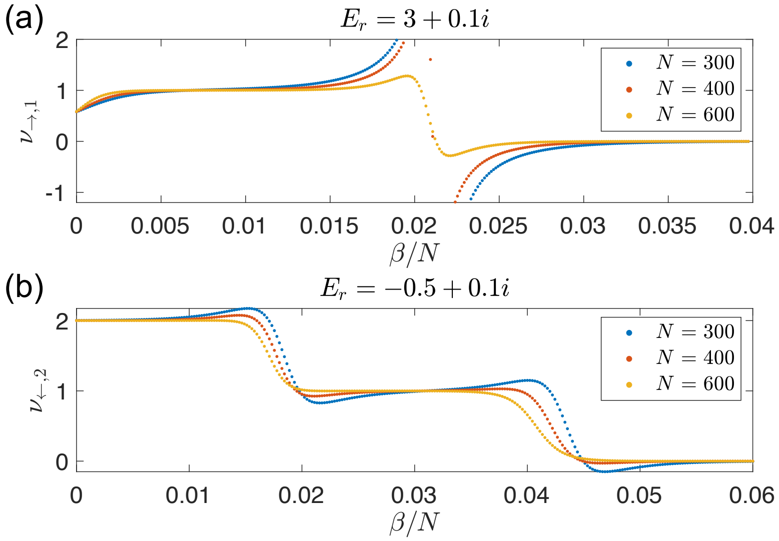

FIG. S2. (a) $\nu_{\rightarrow,1}$ and (b) $\nu_{\leftarrow,2}$ as functions of β/N for the two cases in Fig. S1(b) and (d) respectively, with different numbers of lattice $N = 300$, 400, and 600. As our previous analytical results suggest that the transition value β_c is proportional to N, here the variable β is rescaled by a factor of $1/N$, so as to map the transition points for systems with different sizes to the same parameter. The plateaus are seen to be flatter (hence better quantization) for larger systems. Other parameters are $t_1 = 1$ and $t_{-1} = 0.5$.

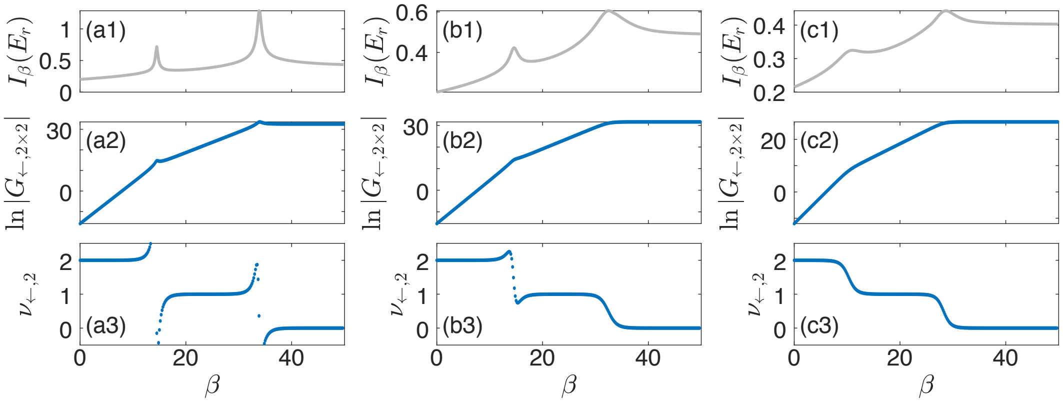

FIG. S3. Disorder effect on the quantized response of the example model of Figs. 1 and 2 in the main text, with the strength of the the disorder given by (a) $W = 0$, (b) $W = 0.2$, and (c) $W = 0.5$. The unperturbed model Hamiltonian is given by Supplementary Eq. **??** with $t_1 = 1$, $t_{-1} = 0.5$, $t_2 = 2$, $t_m = 0$ for every other m, and $N = 100$ lattice sites. Results are obtained with a single choice of the disorder $W_j(x)$ for each panel.

shift the transition value of β, as the shape of the complex spectrum is modified by the disorder. Nevertheless, the quantized plateaus of $\nu_{\rightarrow,2}$ are clearly seen even with a relatively strong disorder with $W = 0.5$ The transitions of $\nu_{\rightarrow,2}$ also match the peaks (or local maximums) of $I_\beta(E_r) = \sum_n |1/(E_n(\beta) - E_r)|$ very well (see Supplementary Eq. **??** in the main text), which indicates the overlapping between the spectrum and the reference energy.

SUPPLEMENTARY NOTE 4: QUANTIZED RESPONSE IN A MULTIBAND SYSTEM

In this section, we extend our discussion to multi-band systems, where there can be more than one spectral loop in the complex energy plane. Since the system is periodic at level of the unit cells, we shall expect topologically quantized response when the Green's function block is chosen appropriately. Suppose we rewrite the Hamiltonian in a basis where the two bands are decoupled, i.e. $H_{\text{e-SSH}} \rightarrow H_1 \bigoplus H_2$ with $1, 2$ the band indices. Since a given reference

energy may now be simultaneously enclosed by different bands with different windings, say E_r is enclosed by band-1 but not by band-2, we shall obtain different quantized responses for different blocks, i.e. a nonzero response only for the block corresponding to H_1, but not that of H_2. On the other hand, in the more experimentally relevant sublattice basis, each sublattice is formed by components from both bands (as each band is formed by both sublattices). Hence we may consider a block of the Green's function given by the elements from one sublattice only, which already contains the winding information of both bands and also takes into account the unit-cell structure of the system.

We demonstrate that this approach gives correct winding number measurements through an illustrative multi-band model, with example computations performed in various parameter regimes (Figs. S4 and S5). Our model is the extended non-Hermitian Su-Schrieffer-Heeger (SSH) [1] with long-range couplings, as described by the Hamiltonian

$$H_{\mathrm{e-SSH}} = \sum_x^N \Big[(t_1+\delta_1)\hat{a}_x^\dagger \hat{b}_x + (t_1-\delta_1)\hat{b}_x^\dagger \hat{a}_x + (t_2\hat{b}_x^\dagger \hat{a}_{x+1} + h.c.) + (t_3+\delta_3)\hat{a}_x^\dagger \hat{b}_{x+2} + (t_3-\delta_3)\hat{b}_{x+2}^\dagger \hat{a}_x\Big] \quad (6)$$

with a and b the two sublattices. The longer-range coupling $t_3 \pm \delta_3$ is to induce large spectral winding number in the complex energy plane, such as to illustrate the validity of our approach for relatively complicated multi-band models with couplings across different distances. The associated momentum-space Hamiltonian is given by

$$H_{\mathrm{e-SSH}}(k) = \begin{pmatrix} 0 & t_1+\delta_1+t_2e^{-ik}+(t_3+\delta_3)e^{2ik} \\ t_1-\delta_1+t_2e^{ik}+(t_3-\delta_3)e^{-2ik} & 0 \end{pmatrix}. \quad (7)$$

In Figs. S4 and S5, we illustrate our approach for several cases with different band structures and spectral winding numbers, and quantized response of m is seen when we consider the $m \times m$ block for only one sublattice, with m the spectral winding number at the chosen reference energy. For example, $G^a_{\leftarrow,3\times3}$ is given by

$$G^a_{\leftarrow,3\times3} = \begin{pmatrix} G_{1(L-5)} & G_{1(L-3)} & G_{1(L-1)} \\ G_{3(L-5)} & G_{3(L-3)} & G_{3(L-1)} \\ G_{5(L-5)} & G_{5(L-3)} & G_{5(L-1)} \end{pmatrix}, \quad (8)$$

with $L = 2N$ and the basis of the matrix is arranged as $(a_1, b_1, a_2, b_2, ..., a_N, b_N)$. In Fig. S5, we illustrate two further examples in different parameter regimes with the two bands being connected and separated respectively. In either case, the maximal spectral winding number is $\nu_{\max}(E_r) = 3$ if we scan over all possible reference energy E_r. Nevertheless, here we choose the reference energy to have $\nu(E_r) = 2$, and the plateaus obtained via using a 2×2 block of the Green's function matrix indeed yields the correct winding number.

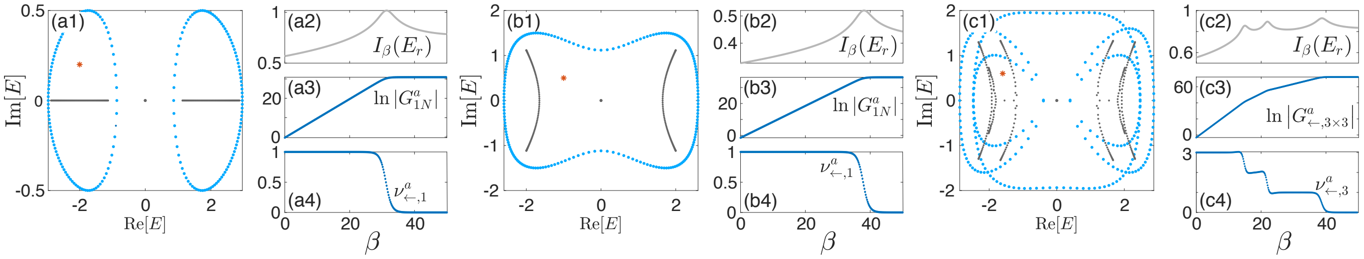

FIG. S4. Quantized response yielding the winding number at E_r for an extended non-Hermitian SSH model with 2 bands and long-range couplings. Blue and gray dots in (a1), (b1), and (c1) are the PBC and OBC spectra respectively, where the red star gives the reference energy E_r. (a) The two PBC bands are separated, each enclosing a nonzero area with a spectral winding number $\nu = 1$. (b) The two PBC bands connects into a single loop with a spectral winding number $\nu = 1$. The quantized response is calculated for the off-diagonal element for a-sublattice only in (a) and (b), with $G^a_{1N} = G^a_{\leftarrow,1\times1}$. (c) The two PBC bands are separated, with a spectral winding number ranging from 1 to 3 for different reference energy E_r enclosed by the spectrum. Here we consider a case with $\nu = 3$, and the quantized response is calculated for the 3×3 off-diagonal block, also for a-sublattice only, as according to Supplementary Eq. 8. Parameters are (a) $t_1 = 1$, $\delta_1 = 0.5$, $t_2 = 2$, $t_3 = \delta_3 = 0$, $N = 100$, and $E_r = -2 + 0.2i$; (b) $t_1 = 1$, $\delta_1 = 1.5$, $t_2 = 2$, $t_3 = \delta_3 = 0$, $N = 100$, and $E_r = -1 + 0.5i$; (c) $t_1 = \delta_1 = 0.5$, $t_2 = 2$, $t_3 = 1$, $\delta_3 = 1.5$, $N = 150$, and $E_r = -1.6 + 0.6i$.

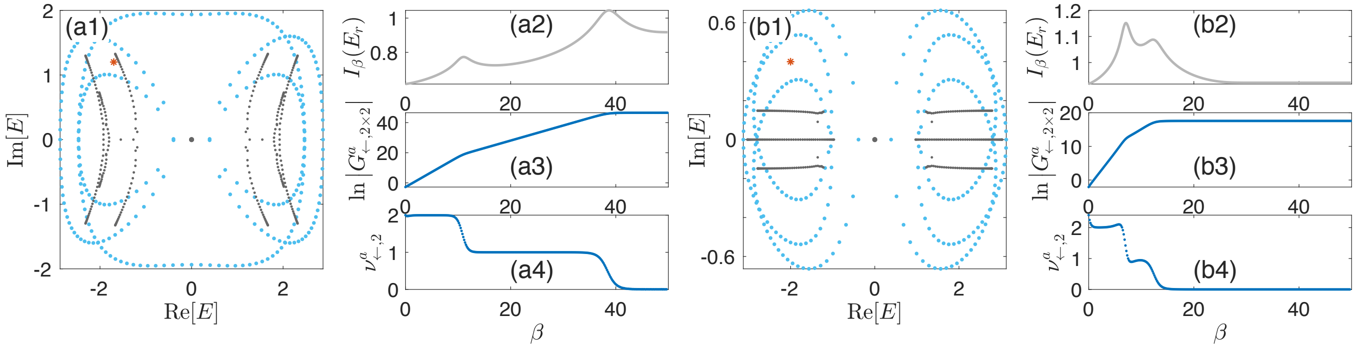

FIG. S5. More examples of classical quantized response yielding the winding number at E_r for an extended non-Hermitian SSH model with 2 bands and long-range couplings. Blue and gray dots in (a1) and (b1) are the PBC and OBC spectra respectively, where the red star gives the reference energy E_r. The two PBC bands are connected into one loop in (a), and separated in (b). In both cases, the maximum spectral winding is 3, and that of the chosen E_r is $\nu(E_r) = 2$. Parameters are (a) $t_1 = \delta_1 = 0.5$, $t_2 = 2$, $t_3 = 1$, $\delta_3 = 1.5$, $N = 150$, and $E_r = -1.7 + 1.2i$. (b) $t_1 = \delta_1 = 0.2$, $t_2 = 2$, $t_3 = 1$, $\delta_3 = 0.5$, $N = 150$, and $E_r = -2 + 0.4i$.

SUPPLEMENTARY NOTE 5: ILLUSTRATIVE DETAILS OF CIRCUIT FOR PROBING THE RESPONSE OF THE HATANO-NELSON MODEL

To be concrete, we provide explicit details for the realization of the simplest case of the circuit representing the Hatano-Nelson model with topological winding $\nu = 1$. It consists of a chain of nodes connected by unbalanced couplings which simultaneously give rise to non-Hermiticity and non-reciprocity. It is well-established that such couplings can be realized with INICs (negative impedance converters with current inversion) [2, 3], which contains operation amplifiers that break the reciprocity. To realize the tuning of the spectral reference point Ω and end-to-end couplings $e^{-\beta(\omega)}$, we also include tunable inductors and additional RLC elements as according to Ref. [4]. Its Laplacian, together with these tunable elements, takes the form

$$J = \Bigg[e^{-\beta(\omega)} \left(e^{\alpha} |N\rangle\langle 0| + e^{-\alpha} |0\rangle\langle N| \right) + \sum_{x=0,\pm}^{N-1} e^{\pm\alpha} |x\rangle\langle x \pm 1| - \sum_{x=0}^{N} \left(2\cosh\alpha - \omega_0^2/\omega^2 + \Omega(\omega) \right) |x\rangle\langle x| \Bigg] \times i\omega C \tag{9}$$

where $\alpha = \tanh^{-1}\frac{C_1}{C_2}$, $C = \sqrt{C_2^2 - C_1^2}$, $\omega_0^{-2} = l_{gr}C$ and $\Omega = \frac{-C_\Omega + iR_\Omega/\omega}{C}$. C_1 and C_2 are capacitors involved in internode couplings as according to Ref. [4], and R_Ω, l_{gr}, C_Ω are RLC elements that connect each node to the ground. By varying the choice of C_Ω and R_Ω, Ω and hence different points of the complex eigenvalue plane can be sampled. In this circuit, the $\nu = 1$ and $\nu = 0$ regions are separated by the curve $\Omega = 2\cosh(\alpha + ik) - 2\cosh\alpha - \omega_0^2/\omega^2$. The effective flux $\beta(\omega)$ can be adjusted by tuning the AC frequency ω, and is given by $\beta(\omega) = \ln[1 - \omega^2 l(C_2 - C_1)]$, where l is an inductor involved in the internode couplings which controls the sensitivity of the tuning. Clearly, by including additional couplings between more distant nodes [5–12], this circuit construction can be extended to models with additional further couplings, such as that shown in the main text with $t_1 = 1, t_{-1} = 0.5, t_2 = 2, t_{-2} = 0$.

* lilh56@mail.sysu.edu.cn
† phylch@nus.edu.sg
‡ phygj@nus.edu.sg

[1] W. P. Su, J. R. Schrieffer, and A. J. Heeger, "Solitons in polyacetylene," Phys. Rev. Lett. **42**, 1698–1701 (1979).
[2] Tobias Hofmann, Tobias Helbig, Ching Hua Lee, Martin Greiter, and Ronny Thomale, "Chiral voltage propagation and calibration in a topolectrical chern circuit," Physical review letters **122**, 247702 (2019).
[3] T Helbig, T Hofmann, S Imhof, M Abdelghany, T Kiessling, LW Molenkamp, CH Lee, A Szameit, M Greiter, and R Thomale, "Generalized bulk–boundary correspondence in non-hermitian topolectrical circuits," Nature Physics , 1–4 (2020).

[4] Linhu Li, Ching Hua Lee, and Jiangbin Gong, "Impurity induced scale-free localization," Communications Physics **4**, 1–9 (2021).

[5] Linhu Li, Ching Hua Lee, and Jiangbin Gong, "Emergence and full 3d-imaging of nodal boundary seifert surfaces in 4d topological matter," Communications physics **2**, 1–11 (2019).

[6] Ching Hua Lee, Amanda Sutrisno, Tobias Hofmann, Tobias Helbig, Yuhan Liu, Yee Sin Ang, Lay Kee Ang, Xiao Zhang, Martin Greiter, and Ronny Thomale, "Imaging nodal knots in momentum space through topolectrical circuits," Nature communications **11**, 1–13 (2020).

[7] Motohiko Ezawa, "Electric circuit simulations of n th-chern-number insulators in 2 n-dimensional space and their non-hermitian generalizations for arbitrary n," Physical Review B **100**, 075423 (2019).

[8] Yuehui Lu, Ningyuan Jia, Lin Su, Clai Owens, Gediminas Juzeliūnas, David I Schuster, and Jonathan Simon, "Probing the berry curvature and fermi arcs of a weyl circuit," Physical Review B **99**, 020302 (2019).

[9] Weixuan Zhang, Deyuan Zou, Jiacheng Bao, Wenjing He, Qingsong Pei, Houjun Sun, and Xiangdong Zhang, "Topolectrical-circuit realization of a four-dimensional hexadecapole insulator," Physical Review B **102**, 100102 (2020).

[10] Ziyin Song, Tianyu Wu, Wenquan Wu, and Rui Yu, "Experimental realization of non-abelian gauge potentials and topological chern state in circuit system," arXiv preprint arXiv:2009.04870 (2020).

[11] Deyuan Zou, Tian Chen, Wenjing He, Jiacheng Bao, Ching Hua Lee, Houjun Sun, and Xiangdong Zhang, "Observation of hybrid higher-order skin-topological effect in non-hermitian topolectrical circuits," arXiv preprint arXiv:2104.11260 (2021).

[12] Alexander Stegmaier, Stefan Imhof, Tobias Helbig, Tobias Hofmann, Ching Hua Lee, Mark Kremer, Alexander Fritzsche, Thorsten Feichtner, Sebastian Klembt, Sven Höfling, *et al.*, "Topological defect engineering and p t symmetry in non-hermitian electrical circuits," Physical Review Letters **126**, 215302 (2021).
